# Erythema Sweetobullosum: A Reactive Cutaneous Manifestation of Coccidioidomycosis

**DOI:** 10.1177/2324709618796659

**Published:** 2018-08-24

**Authors:** Hisham Abukamleh, Arash Heidari, Greti Petersen, Piruthiviraj Natarajan, Gian Yakoub, Everardo Cobos, Royce Johnson

**Affiliations:** 1 Kern Medical Center, Bakersfield, CA, USA

**Keywords:** coccidioidomycosis, cutaneous manifestation, erythema sweetobullosum, noninfectious

## Abstract

Reactive cutaneous coccidioidal skin manifestations are commonly noticed during the early stage of coccidioidomycosis. These skin lesions are devoid of any active coccidioidal organism, and the immune trigger mechanisms are not elucidated. We describe 6 cases of unusual reactive cutaneous coccidioidal manifestation, characterized by painful vesiculobullous lesions known as erythema sweetobullosum. The biopsy of the lesions revealed neutrophilic dermatosis with inflammatory cells resulting in a cleft and elevation of the most superficial layer of the skin forming a bulla. The reactive cutaneous lesion is self-limited and requires no specific therapy.

## Background

Immune-mediated skin reactions to coccidioidomycosis manifest commonly as erythema nodosum and erythema multiforme.^1^ A less well known noninfectious cutaneous manifestation of coccidioidomycosis is termed erythema sweetobullosum (ESB). This manifests as vesiculobullous eruptions predominantly found in upper arms and contiguous chest and was named erythema sweetobullosum by dermatologist Dr David J. Elbaum in 1998.^2^

Coccidioidomycosis is caused by *Coccidioides immitis* and *posadasii*, which result in an identical spectrum of illness. The disease is endemic to Southwestern United states, Northwestern Mexico, Central America, Brazil, and Argentina. There is a new endemic cluster in eastern Washington state.^3^ Annually, 150 000 infections are estimated to occur in the United States.^3,4^ Approximately 60% of the infected are asymptomatic. Roughly 40% of the infected are symptomatic, and only 10% of them are diagnosed.^4,5^ The primary pulmonary infection typically presents as community-acquired pneumonia. About 1% of the infected develop disseminated disease, pathologically exhibiting granulomatous lesions with endosporulating spherules. Reactive cutaneous manifestations can be seen in up to 50% of the primary pulmonary infections.^1,6,7^

## Case Presentation

### Case 1

**Figure 1.**
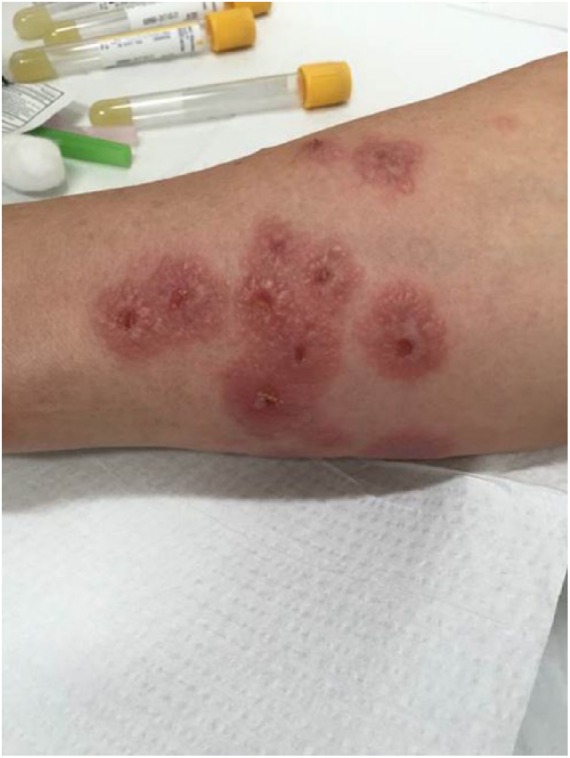
Drained bullous lesions on the right leg.

**Figure 2.**
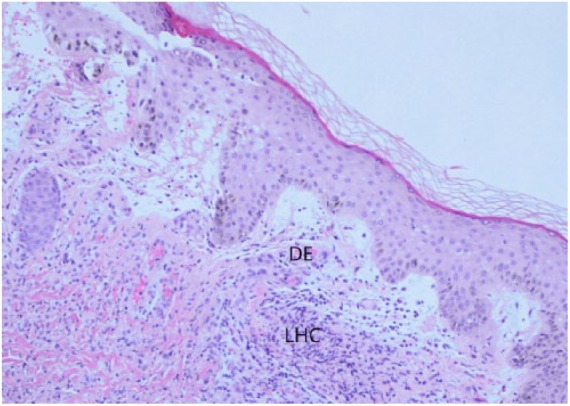
H&E at 10X magnification. DE, dermal edema; LHC, lymphohistiocytic inflammatory cells.

A 40-year-old female presented at the emergency department with painful rash associated with intermittent fever and joint pain for 5 days (Figure 1). The rash was recognized initially over the neck and bilateral upper extremities, which subsequently spread to the legs. Multiple tense raised vesicles and bullous lesions were noticed bilaterally over the forearm and arm with several lesions associated with drainage and others crusted. Some of the bullous lesions had surrounding erythema. Laboratory examination revealed a white blood cell (WBC) count of 15.5 × 10^3^/µL with an absolute eosinophil count (AEC) of 1600 cells/µL. The coccidioidal serology was positive by immunodiffusion for immunoglobulin M antibody (ID-IgM). The complement fixation (CF) antibody titer was <1:2. Chest X-ray revealed right upper lobe infiltrate. She received fluconazole 800 mg daily, and subsequent follow-up showed a nonreactive ID-IgM but weakly reactive ID-IgG and CF titer of 1:2. The punch biopsy of the drained bullous lesions of the right leg (Figure 1) showed superficial dermal edema enriched with lymphohistiocytic inflammation (Figure 2). The periodic acid–Schiff stain and Gomori methenamine silver nitrate stain *were* negative for fungal organisms.

### Case 2

**Figure 3.**
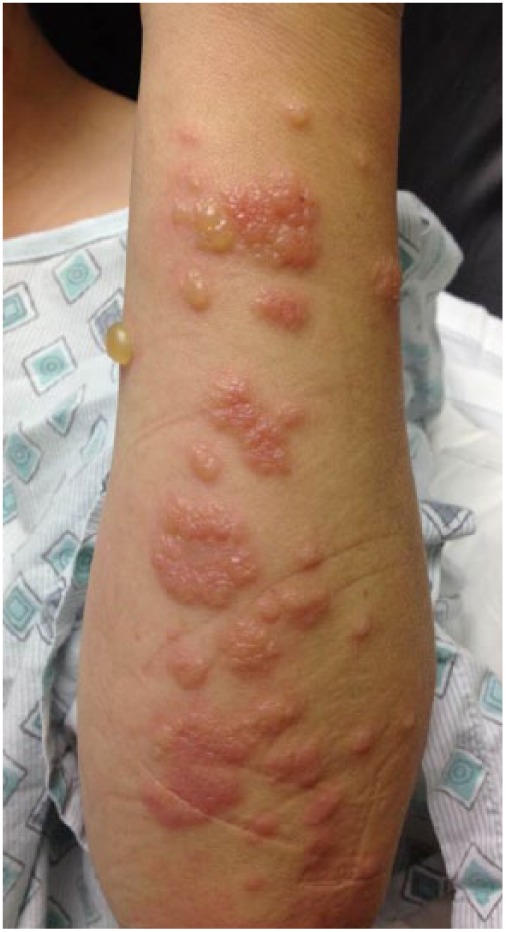
Bullous lesions on the forearm with raised plaques.

A 45-year-old female presented with skin rash for 8 days (Figure 3). Rash was initially noticed on the right arm, which spread to the left arm and trunk. The patient complained of nonproductive cough, arthralgia, and weight loss. On examination, vesiculobullous, pruritic rashes with tenderness around the lesions were noticed. Biopsy of the forearm bullae lesion revealed histiocytes and neutrophils in the subepidermal layers with break in the epithelial lining. She received fluconazole 400 mg. Laboratory examination revealed a WBC count of 12.1 × 10^3^/µL with an AEC of 1100 cells/µL. The coccidioidal serology showed weakly reactive ID-IgM and ID-IgG. CF antibody titer was 1:2. One month later she returned to the clinic with complete resolution of the rash.

### Case 3

**Figure 4.**
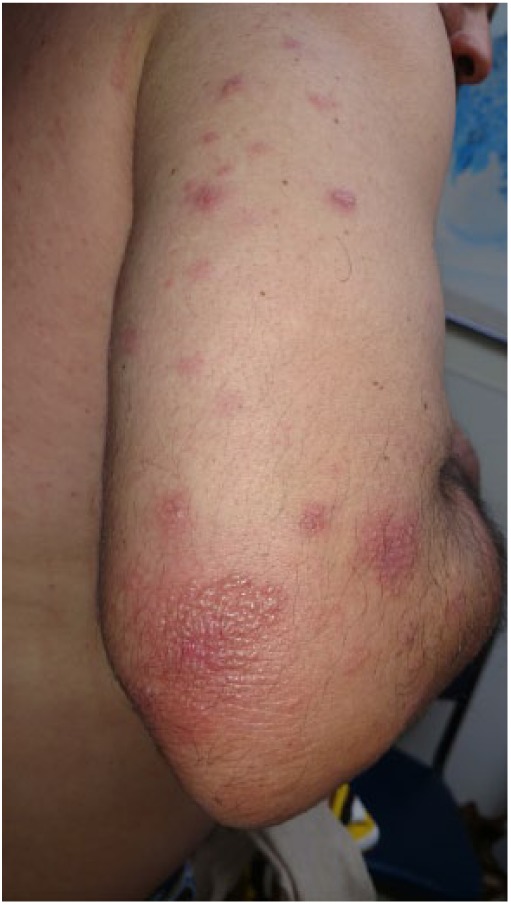
Vesicles and bullous lesion on the arm.

**Figure 5.**
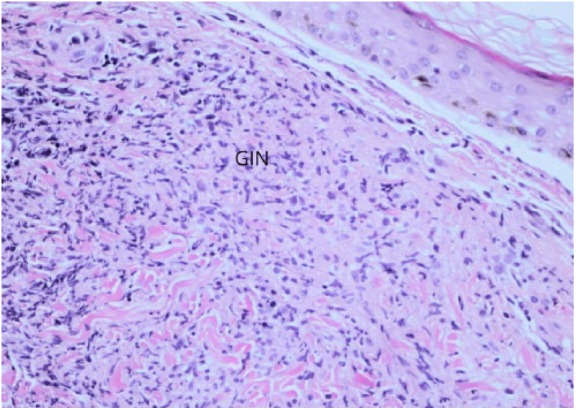
H&E at 20X magnification. GIN, granulomatous inflammatory cells.

A 47-year-old female presented with skin rash for 17 days (Figure 4). Initially the vesicular rash involved both forearms, which subsequently spread to the neck and both legs. The rash was notable for pruritic erythema around vesicular lesions. Biopsy of the lesions in the neck revealed granulomatous inflammation in the dermis (Figure 5). Laboratory tests revealed WBC count of 12.7 × 10^3^/µL and AEC of 900 cells/µL. The coccidioidal serology was weakly reactive for ID-IgM and ID-IgG, and CF antibody titer was <1:2. Chest X-ray showed small right lower lobe infiltrate. The rash resolved in the next 2 weeks.

### Case 4

**Figure 6.**
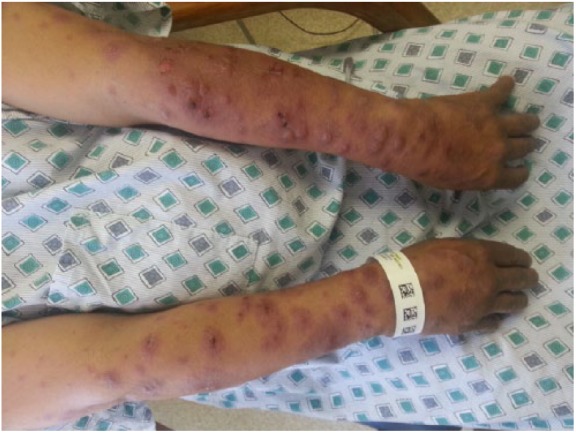
Vesiculo-bullous lesion on the bilateral forearms.

**Figure 7.**
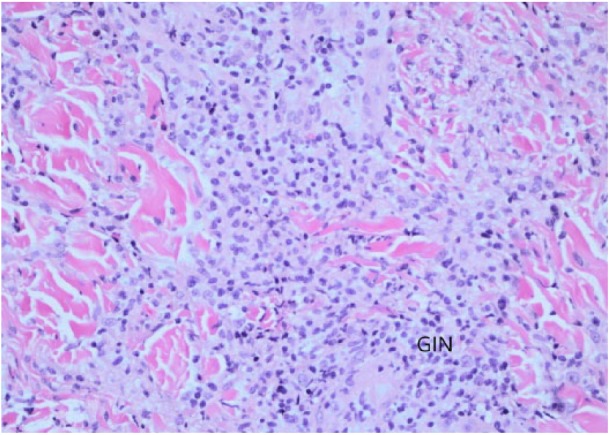
H&E at 20X magnification. GIN, granulomatous inflammatory cells.

A 42-year-old male presented with painful, pruritic vesiculobullous rash on his bilateral forearms for 15 days (Figure 6). The rash was red, raised, tense and vesiculobullous, and tender. He complained of fever and cough. Biopsy of the left forearm lesion revealed granulomatous inflammatory cells in the dermis with minimal subepidermal edema (Figure 7). The laboratory examination revealed WBC count of 12.3 × 10^3^/µL and AEC of 800 cells/µL. The coccidioidal serology showed reactive ID-IgM and ID-IgG with CF antibody titer <1:2. The chest X-ray showed right lower lobe infiltrate. The skin biopsy showed subepidermal vesicular dermatitis with neutrophils and histiocytes. In the next 2 weeks, the rash resolved completely.

### Case 5

**Figure 8.**
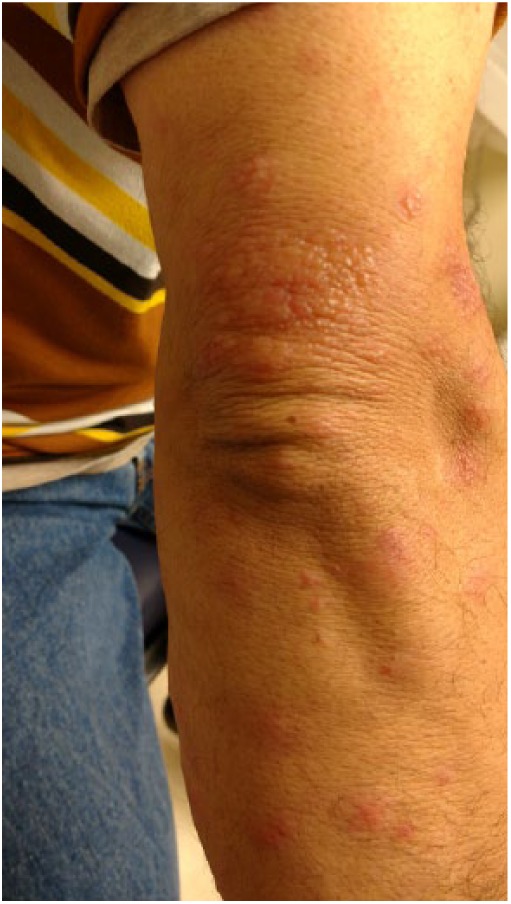
Vesiculo-bullous lesions on the arm.

**Figure 9.**
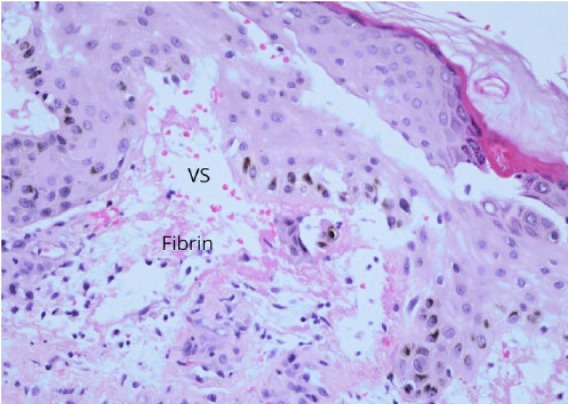
H&E at 20X magnification. VS, vesiculated subepidermal layer.

A 45-year-old male presented with fever and diffuse maculopapular rash that started on bilateral forearms then over 4 days spread to lower extremities, shoulder, and posterior thorax (Figure 8). He complained of dry cough and mild pruritus over the chest. The laboratory examination showed WBC count of 16.7 × 10^3^/µL with an AEC of 1600 cells/µL. Biopsy of the lesions in the posterior right arm revealed dermal edema and subepidermal vesicle with fibrin and irregular brown pigmentation in the epidermis (Figure 9). The coccidioidal serology showed weakly reactive ID-IgM and ID-IgG, and CF antibody titer was <1:2. Chest X-ray showed bilateral infiltrates worse on the left lower lobe. The rash resolved in the next 2 weeks.

### Case 6

**Figure 10.**
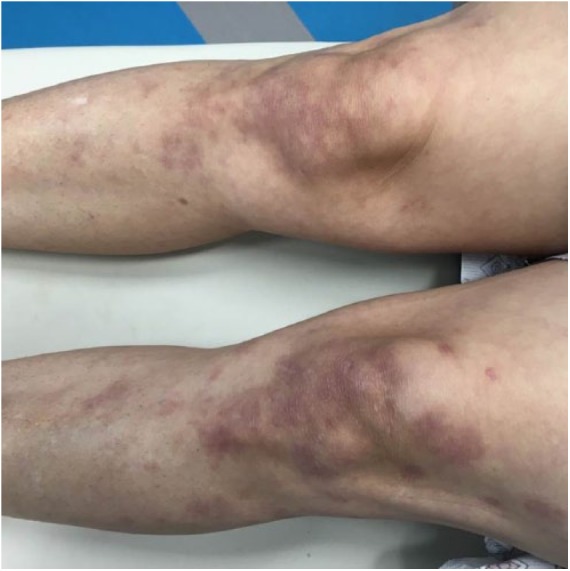
Bilateral crusted bullous lesion the leg and knee.

**Figure 11.**
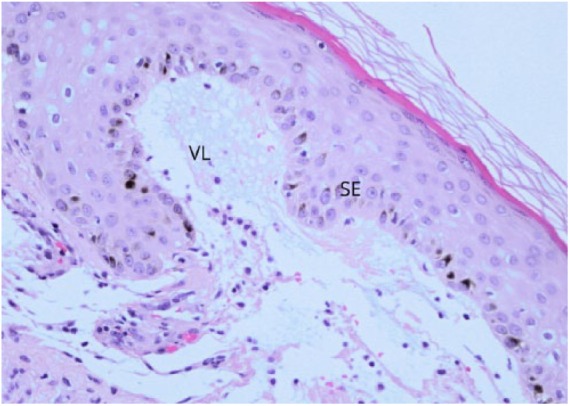
H&E at 20X magnification. VL, vesiculated layer; SE, subepidermal edema.

A 27-year-old Caucasian male presented with rash associated with myalgia and fever for 7 days (Figure 10). Multiple open and intact vesicles associated with erythematous subcutaneous tender nodules on both arms, legs, neck, and forehead were noted. There was clear, serous discharge from the vesicles. Biopsy of the crusted lesion over the left knee revealed vesiculated subepidermal layer with histiocytic inflammation of the dermis (Figure 11). Laboratory examination showed WBC count of 11.7 × 10^3^/µL with an AEC of 1200 cells/µL. The chest X-ray showed right upper lobe inflammatory infiltration. The coccidioidal serology showed reactive ID-IgM and weakly reactive ID-IgG, and CF antibody titer was <1:2. The patient was discharged but lost to follow-up (see Table 1).

**Table 1.** Summary of Laboratory Reports.

Case	Age/Sex	Date of Presentation After Skin Lesion	Lesion Distribution	Biopsy	White Blood Cells	Absolute Eosinophilic Count	CF Titer
1	40/female	5 Days	Neck, both arms subsequently to legs	Superficial dermal edema enriched with lymphohistiocytic inflammation	15.5 × 10^3^/µL	1600 cells/µL	<1:2
2	45/female	8 Days	Right arm to left arm and trunk	Neutrophils and histiocytes in the subepidermal layers with break in the epithelial lining	12.1 × 10^3^/µL	1100 cells/µL	1:2
3	47/female	17 Days	Both forearms spreading to legs and neck	Granulomatous inflammation of the dermis	12.7 × 10^3^/µL	900 cells/µL	<1:2
4	42/male	15 Days	Both forearms	Granulomatous inflammation of the dermis with minimal subepidermal edema	12.3 × 10^3^/µL	800 cells/µL	<1:2
5	45/male	4 Days	Bilateral forearm, upper back, shoulder, and both legs	Subepidermal vesicle with fibrin	16.7 × 10^3^/µL	1600 cells/µL	<1:2
6	27/male	7 Days	Both arms, legs, neck, and forehead	Vesiculated subepidermal layer with histiocytic inflammation on the dermis	11.7 × 10^3^/µL	1200 cells/µL	<1:2

Abbreviation: CF, complement fixation.

Laboratory results are summarized in Table 1.

## Discussion

The skin manifestation of coccidioidal infection are broadly classified as reactive cutaneous and organism-specific manifestations.^1^ The organism can be demonstrated at the site of the lesion with organism-specific manifestations whereas the reactive cutaneous manifestation is usually a delayed-type immune reaction.^1,8^ The various reactive manifestations of *Coccidioides* are erythema nodosum, erythema multiforme, and ESB. Different type of cutaneous cell layer involvement results in different types of cutaneous manifestation. The exact immune triggers in each of these entities are largely unknown. A benign or asymptomatic coccidioidal infection results in a significant delayed-type immune response in the host associated with low or nondemonstrable complement fixing antibodies.^9^ Conversely, a profound and multifocal infection is usually noted to have high CF antibodies and low delayed-type hypersensitivity.^10^ All our patients had high absolute eosinophil levels. The reactive cutaneous manifestations occur very early in the disease and self-resolves within weeks. A biopsy of the bullous lesion usually shows dermatitis with epidermal layers infiltrated with eosinophil and phagocyte-rich stroma suggesting the inflammation lifting the most superficial layers of the skin producing the bullae. Similar histology was associated with vesicular lesions. The histologic features appear to change during the course, resulting in varied histologic findings within our cohort. Early biopsy showed features of lymphocyte-rich epidermal tissues with tissues edema and necrosis. It is followed by a distinct neutrophil predominant phase as it progresses. Macrophages with persistent tissue edema were noticed in older lesions. In the resolving phase, granulomatous features were noticed without demonstrable organism at these lesions. Even though the bullous lesions were extensive they regressed within few weeks, and there is no known role for corticosteroids to reduce the inflammation. The bullae can rupture and may require local care.

## Conclusion

Erythema sweetobullosum is an inflammatory skin reaction presenting as clusters of painful, well-demarcated papules with central vesicular/bullae formation. It is more commonly found on arms but can be seen on lower extremities and trunk. It manifests in acute coccidioidomycosis infection and usually resolves in a short period without long-term sequelae. If there is a concern for the diagnosis, a skin biopsy may be of assistance.
